# Fecal microbiota transplantation for recurrent C. difficile infection in patients with inflammatory bowel disease: experience of a large-volume European FMT center

**DOI:** 10.1080/19490976.2021.1994834

**Published:** 2021-10-28

**Authors:** Gianluca Ianiro, Stefano Bibbò, Serena Porcari, Carlo Romano Settanni, Federica Giambò, Andreea Roxana Curta, Gianluca Quaranta, Franco Scaldaferri, Luca Masucci, Maurizio Sanguinetti, Antonio Gasbarrini, Giovanni Cammarota

**Affiliations:** a Digestive Disease Center, Fondazione Policlinico Universitario A. Gemelli IRCCS, Università Cattolica Del Sacro Cuore, Rome, Italy; b Microbiology Unit, Fondazione Policlinico Universitario “A. Gemelli” IRCCS, Università Cattolica Del Sacro Cuore, Rome, Italy

**Keywords:** Fecal microbiota transplantation, Clostridioides difficile infection, inflammatory bowel disease, gut microbiota, microbiome, ulcerative colitis, Crohn’s disease

## Abstract

Inflammatory bowel disease (IBD) is a risk factor for *C. difficile* infection (CDI), which, in turn, complicates the clinical course of IBD. Fecal microbiota transplantation (FMT) is safe and effective in patients with IBD and recurrent CDI (rCDI). In our study, patients with IBD and rCDI received FMT by colonoscopy and were followed-up for 8 weeks. The primary outcome was negative *C. difficile* toxin 8 weeks after FMT. Eighteen patients with IBD were enrolled. Eight patients received sequential FMT either for pseudomembranous colitis or failure of single fecal infusion. At 8-week follow-up the *C. difficile* toxin was negative in 17 patients, and most (83%) experienced also improvement of IBD disease activity. Overall, we did not observe any serious adverse event.

FMT appears to be highly effective and safe in patients with IBD and rCDI and is likely not only to eradicate CDI but also to improve disease activity of IBD.

## Introduction

*Clostridioides difficile* infection (CDI) is the most common healthcare-associated infectious disease.^1,2^ Inflammatory bowel disease (IBD) is associated with higher prevalence, recurrence, and severity of CDI.^3–6^ Moreover, CDI superinfection in patients with IBD is associated with increased rate of hospitalizations, escalation of IBD therapy, length of hospital stay, colectomy, death, and health-care costs.^7,8^

Fecal microbiota transplantation (FMT) is an established therapy for recurrent CDI (rCDI),^9,10^ as recommended by international guidelines.^11,12^ Increasing evidence suggests that FMT is safe and effective in patients with IBD and rCDI, improves disease activity and reduces the need for escalation of IBD therapy.^13–16^ Most data come from the U.S., and, to our best knowledge, there are no reports from Europe. Our aim is to report outcomes of patients with IBD treated with FMT for rCDI in a large-volume European FMT center.

## Methods

### Study design and patients

This is a sub-analysis of patients with IBD from a single-center, prospective cohort study,^17^ reported following STROBE guidelines.^18^ We considered for inclusion all patients with confirmed diagnosis of IBD referred to our FMT center for rCDI from July 2016 to January 2021. Exclusion criteria were history of bowel resection, pregnancy, breastfeeding, concomitant treatment with bezlotoxumab, and enrollment in other clinical trials. All included subjects provided their written informed consent.

The primary outcome was negative *C. difficile* toxin at 8 weeks after FMT regardless clinical symptoms. Secondary outcomes were IBD activity and safety of FMT at 8-week follow-up.

### Baseline assessments

Baseline IBD activity was assessed through Harvey-Bradshaw Index (HBI)^19^ for Crohn’s disease (CD) and partial Mayo score^20^ for ulcerative colitis (UC]). IBD was considered to be active if HBI ≥4 and partial Mayo score ≥2, while clinical remission was identified by HBI <4 and partial Mayo score <2. Time of IBD diagnosis, concomitant IBD therapy, number of CDI recurrences and previous treatments for CDI were also collected. Endoscopic disease activity was evaluated at the time of FMT through the endoscopic Mayo score^21^ for UC and the simple endoscopic score (SES)-CD for CD.^22^ The severity of CDI was assessed following international guidelines.^23^

### Interventions and follow-up

Donor selection, manipulation of feces and FMT delivery were performed as previously described^17^ for patients enrolled until May 2020, while we applied specific measures to prevent COVID-19 diffusion for patients enrolled afterward.^24,25^ All procedures were done by colonoscopy. Each patient received at least one fecal transplant. Moreover, we repeated FMT in specific clinical conditions. A sequential FMT protocol was applied *a priori* to hospitalized patients, those with severe CDI, and those with endoscopic evidence of pseudomembranous colitis (PMC), as these variables have been identified as predictors of early failure after FMT.^26,27^

All these patients were scheduled for at least two fecal infusions, and those with PMC underwent FMT until the disappearance of pseudomembranes, as already experienced.^10^

Each fecal infusion was administered every 3 days, and patients were also restricted to a light diet and underwent a restricted bowel preparation (2 liters of macrogol) before each procedure.

Moreover, if patients reported diarrhea after FMT, *C. difficile* toxin was repeated and, if positive, further fecal infusions were administered until the resolution of diarrhea and negativization of *C. difficile* toxin.

Patients were followed up at week 8 after last FMT or earlier in case of symptom flares. At each visit, IBD activity was assessed through HBI and partial Mayo score, and adverse events were recorded.

## Results

Characteristics of patients are detailed in Table 1. In the study period 25 patients with IBD received FMT for rCDI, but seven were excluded because of prior subtotal colonic resection (n = 1) or concomitant participation to another clinical trial (n = 6). Eighteen patients (mean age 50 years old [range 21–79], 8 females) were included in the final analysis. Sixteen subjects had UC and two had CD. The median time from the diagnosis of IBD was 5 years (range: 1–30). Among patients with UC, two presented with proctitis, six with left-sided colitis, and eight with pancolitis. The median partial Mayo score was 6, and the disease activity was mild in two patients, moderate in 12 patients and severe in two patients. The endoscopic disease activity was mild in three patients, moderate in 10 patients, and severe in three patients.

**Table 1.** Characteristics of patients at baseline, of treatments, and of outcomes after FMT

	N
Baseline characteristics of patients	
Total number of patients	18
Males/females	10/8
Median age (range)	50 (21–79)
Median time (years) from IBD diagnosis (range)	5 (1–30)
Ulcerative colitis	16
Location	
E1 (proctitis)	2
E2 (left sided)	6
E3 (pancolitis)	8
Crohn’s disease	2
Location	
L1 (ileal)	1
L2 (colonic)	0
L3 (ileocolonic)	1
L4 (upper GI tract)	0
Phenotype	
B1 (inflammatory)	1
B2 (stricturing)	1
B3 (penetrating)	0
IBD Therapies	
Systemic 5-ASA	12
Topic 5-ASA	6
Systemic corticosteroids	5
Topic corticosteroids	5
Immunosuppressants	1
Biologics	7
Clinical activity of disease at baseline	
Ulcerative colitis (partial Mayo Score)	
Remission	0
Mild	3
Moderate	10
Severe	3
Crohn’s disease (Harvey-Bradshaw index)	
Remission	0
Mild	0
Moderate	2
Severe	0
Endoscopic activity of disease at baseline	
Ulcerative colitis (endoscopic Mayo Score)	
Remission	0
Mild	3
Moderate	10
Severe	3
Crohn’s disease (SES-CD)	
Remission	0
Mild	1
Moderate	0
Severe	1
Antibiotic treatments before FMT	
Vancomycin	18
Metronidazole	5
Fidaxomicin	2
Median number of CDI recurrences (range)	2 (1–5)
Outpatients/inpatients	16/2
Clinical picture of CDI	
Mild	16
Severe	2
Pseudomembranous colitis	3
**Treatments**	
Donors	
Unrelated	18
Related	0
Number of fecal infusions	
N = 1	9
N = 2	8
N = 3	1
**Post-FMT outcomes**	
Eradication of CDI (negative toxin)	
After single fecal infusion	9/15
After multiple fecal infusion (post-FMT failure)	5/6
After *a priori* sequential FMT	3/3
Overall	17/18
Clinical activity of disease after FMT	
Ulcerative colitis (partial Mayo Score)	
Remission	9
Mild	3
Moderate	4
Severe	0
Crohn’s disease (Harvey-Bradshaw index)	
Remission	1
Mild	1
Moderate	0
Severe	0
Serious advent events	0

Among patients with CD, one had ileal disease and another one had ileocolonic involvement with stenosis of the sigmoid colon. Both patients presented with moderate disease activity, and the median HBI was 8. At endoscopic evaluation, one patient had mild and another one severe disease activity. Twelve patients were on systemic salicylates, six on topic salicylates, five on systemic corticosteroids, five on topic corticosteroids, one on azathioprine, and seven on biologics (infliximab, adalimumab, golimumab, or vedolizumab) at the time of our evaluation.

All patients reported worsening of their clinical picture after the infection. The median number of CDI recurrences was 2 (range: 1–5). Before FMT, patients had been treated with vancomycin (n = 18), metronidazole (n = 5) and fidaxomicin (n = 2). Two patients were hospitalized for severe CDI while 16 patients presented with mild CDI and received FMT as outpatients. PMC was found at endoscopy in three patients.

All patients underwent at least one fecal infusion. The three patients with PMC received *a priori* multiple fecal infusions until the disappearance of pseudomembranes (two infusions in two patients, and three infusions in the other patient). Six of the remaining 15 patients (40%) received a further fecal infusion for persistence of diarrhea and of *C. difficile* toxin between 7 and 10 days after the first FMT. All patients received frozen fecal infusions from unrelated donors.

**Figure 1.**
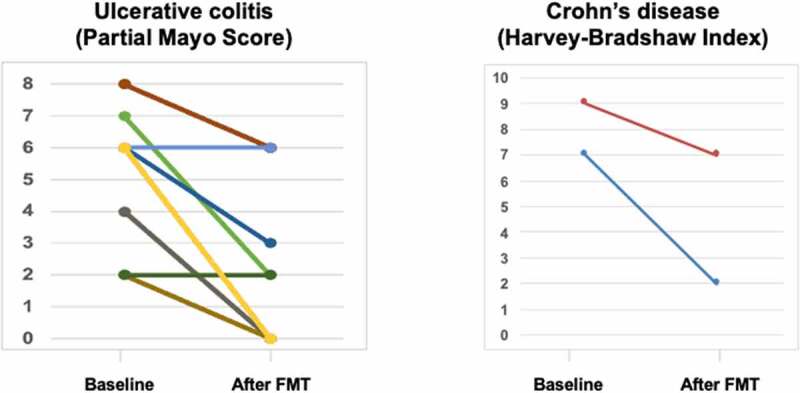
Disease activity indexes before and after FMT in our cohort

At 8-week follow-up, the *C. difficile* toxin was negative in 17 patients (94%), and most of them experienced improvement of clinical picture: 10 patients were on clinical remission (59%) and in four patients (24%) we observed an amelioration of disease activity (from moderate to mild activity, [n = 2] and from severe to moderate activity [n = 2]), while the disease activity did not improve in three patients despite CDI decolonization. Median HBI scores decreased from 8 before FMT to 4 after FMT (*p* = .3), while the median partial Mayo score decreased from 6 before FMT to 0 after FMT (*p* = .0017) (Figure 1). In one patient with CD and stenosis of the sigmoid colon, *C. difficile* toxin and diarrhea persisted despite two fecal infusions. As the narrowing prevented to infuse feces in the cecum, we stopped FMT and treated him with fidaxomicin. Overall, we did not observe any serious adverse event, and the treatment was well tolerated.

## Discussion

In our cohort of patients with IBD, FMT achieved similar cure rates of rCDI (94%) as those previously observed in the general population,^9^ and the procedure was well tolerated. Our data confirm previous observations,^13–16^ and pinpoint specific considerations that may improve the management of patients with IBD and rCDI.

First, as already pointed out by Allegretti and colleagues,^16^ patients with IBD can complain diarrhea regardless CDI, so the decolonization of *C. difficile*, rather than the disappearance of diarrhea, should be used to assess FMT efficacy in clinical practice.

Our experience confirms that CDI can complicate the course of IBD, as 89% of patients presented with moderate or severe disease, and probably their clinical conditions worsened because of the infection. As IBD is a risk factor for CDI recurrence,^28^ the provision of sustained CDI cure by FMT is particularly relevant in this population. In our cohort, most patients who cleared CDI experienced also clinical remission (59%) or improvement of disease activity (24%). Cost-effectiveness studies aimed at assessing the overall benefit of FMT as treatment for CDI in patients with IBD are advocated.

Interestingly, our data suggest that sequential FMT may be adopted as tailored protocol in this subpopulation, for several reasons. First, patients with IBD are more likely to experience severe CDI,^4–6^ and sequential FMT is known to improve efficacy rates of FMT in this clinical setting.^10,29^ In our cohort, all patients with severe CDI were cured by repeat FMT, confirming our previous data^10^ also in this population. Moreover, sequential FMT can improve eradication rates of rCDI.^10^ In our cohort, CDI recurred in 38% of patients who received initially a single fecal infusion, and a second infusion was effective in 83% of them. We did not succeed in one patient with CD and a narrowing of the sigmoid colon, probably because it was not possible to colonize the whole colon with healthy donor feces. This hypothesis is supported also by the low efficacy rates of FMT enemas, that are limited to the left/sigmoid colon.^9^ Other routes, including capsules or nasoduojejunal tube, may potentially be useful in such cases, but their efficacy needs to be proven yet. The potential reasons for higher efficacy of sequential FMT over single FMT in this and other clinical settings (i.e., donor microbiota engraftment) are also yet to be clarified and deserve further investigations.

The main limitation of our study is the small sample, that prevented us to assess predictors of failure through multivariate analysis. Nevertheless, its single-center design allows to study a homogeneous population and avoid biases associated with mixed treatment protocols. Larger, multicenter studies that allow a multivariate analysis of predictors of FMT failure are advocated to improve the efficacy rates of FMT in this population.

Finally, also the role of targeted and reproducible microbial consortia in this setting of patients warrants further investigation, as current data are conflicting.^30,31^

In conclusion, in our cohort of patients with IBD, FMT achieved the same excellent cure rates of CDI already observed in the general population, together with a high safety profile. Although the role of therapeutic manipulation of microbiome in IBD is still not established,^32^ increasing evidence, including our study, suggests that FMT is ready to be used routinely in clinical practice in patients with IBD and rCDI.

Our data also suggest that repeat of fecal infusions can increase the efficacy rates of FMT, and that a sequential protocol should be considered *a priori* in this population to improve the cure rates of CDI, and to reduce the disease activity of IBD.

## Data Availability

The data underlying this article will be shared on reasonable request to the corresponding author.
